# Costs of antibiotic resistance – separating trait effects and selective effects

**DOI:** 10.1111/eva.12187

**Published:** 2014-07-14

**Authors:** Alex R Hall, Daniel C Angst, Konstanze T Schiessl, Martin Ackermann

**Affiliations:** 1 Institute of Integrative Biology, ETH ZürichZürich, Switzerland; 2 Institute of Biogeochemistry and Pollutant Dynamics, ETH ZürichZürich, Switzerland; 3 Department of Environmental Microbiology, Swiss Federal Institute of Aquatic Science and Technology (Eawag)Dübendorf, Switzerland

**Keywords:** antibiotic resistance, cost of resistance, epistasis, experimental evolution, genotype-by-environment interaction

## Abstract

Antibiotic resistance can impair bacterial growth or competitive ability in the absence of antibiotics, frequently referred to as a ‘cost’ of resistance. Theory and experiments emphasize the importance of such effects for the distribution of resistance in pathogenic populations. However, recent work shows that costs of resistance are highly variable depending on environmental factors such as nutrient supply and population structure, as well as genetic factors including the mechanism of resistance and genetic background. Here, we suggest that such variation can be better understood by distinguishing between the effects of resistance mechanisms on individual traits such as growth rate or yield (‘trait effects’) and effects on genotype frequencies over time (‘selective effects’). We first give a brief overview of the biological basis of costs of resistance and how trait effects may translate to selective effects in different environmental conditions. We then review empirical evidence of genetic and environmental variation of both types of effects and how such variation may be understood by combining molecular microbiological information with concepts from evolution and ecology. Ultimately, disentangling different types of costs may permit the identification of interventions that maximize the cost of resistance and therefore accelerate its decline.

## Introduction

Bacterial resistance to antibiotics impairs our capacity to treat infections, posing a growing challenge for global public health (Levy and Marshall 2004; Bergstrom and Feldgarden 2008; Smith and Coast 2013). From the bacterial perspective, resistance is highly advantageous in the presence of antibiotics. However, resistance mechanisms can also impair cellular functions, in turn affecting phenotypic traits such as growth and survival relative to sensitive genotypes in the absence of antibiotics (‘trait effects’ – Box 1). These trait effects can lead to changes in allele frequencies over time (‘selective effect’ – Box 1). For example, if a resistance mutation or plasmid causes bacteria to grow and divide at a slower rate in the absence of antibiotics, this can result in a decrease in the frequency of bacteria with this genotype in a population. Thus, selective effects can be quantified experimentally by monitoring genotype frequencies in populations containing bacteria with and without resistance alleles (Lenski et al. 1991; Trindade et al. 2009; Chevin 2011; Gullberg et al. 2011). In many studies, costs of resistance are defined more loosely, often referring to an effect on a particular phenotypic trait, such as doubling time during exponential growth or stationary phase population density in pure culture (Nagaev et al. 2001; Nilsson et al. 2006; MacLean and Buckling 2009; Paulander et al. 2009; Petersen et al. 2009; Hall et al. 2011). Here, we refer to a cost of resistance strictly as an effect on allele frequencies, that is, a selective effect rather than a trait effect. The motivation for this review is the notion that a clearer distinction between effects on individual traits such as growth rate or yield (trait effects) and how those effects translate to changes of allele frequencies over time (selective effects) may improve our ability to explain variation of costs of resistance.

The selective effect of resistance alleles in drug-free conditions is a key determinant of the long-term stability of resistance in pathogenic populations (Andersson and Levin 1999; Levin 2001; Andersson 2003; Cohen et al. 2003; Andersson and Hughes 2010). For simplicity, we focus on costs of resistance in drug-free conditions, but note that a complete understanding of selection on resistance alleles would incorporate their selective effects across a wide range of drug concentrations, because drug concentrations in nature may vary continuously, rather than categorically, over space and time (Baquero and Negri 1997; Hermsen et al. 2012). Improved understanding of selective effects potentially enables better management of resistance. For example, if resistance is under negative selection in the absence of drugs, a simple way to reduce resistance would be to reduce antibiotic consumption. However, in cases where use of specific antibiotics has been scaled back, resistance sometimes declines and sometimes does not (Seppälä et al. 1997; Enne et al. 2001; Arason et al. 2002; Bean et al. 2005; Gottesman et al. 2009; Sundqvist et al. 2010; Schechner et al. 2013). This indicates that selective effects of resistance are variable. Consistent with this, experiments show that both trait effects and selective effects – and thus the costs of resistance – vary depending on environmental factors (Table 1). For example, the same resistance mutation may be under negative selection in one type of antibiotic-free growth medium and positive selection in another (Trindade et al. 2012). This makes it difficult to predict the selective effect of a given resistance mechanism outside the laboratory. Trait effects and their resultant selective effects can also vary depending on other alleles in the same genetic background, such as those conferring resistance to other antibiotics (Trindade et al. 2009; Andersson and Hughes 2011), compensatory mutations that epistatically buffer the effects of resistance alleles (Schrag et al. 1997; Levin et al. 2000; Reynolds 2000; Maisnier-Patin et al. 2002, 2007; Maisnier-Patin and Andersson 2004; Kim and Wei 2007; Hall et al. 2010), or regulatory mechanisms that alter the expression of resistance mechanisms in different conditions (Martínez and Rojo 2011).

**Table 1:** Environmental variation of trait (growth rate or growth yield measured by pure culture assays) and selective (competitions) effects. Each study demonstrates variation of the effects of resistance alleles depending on experimental conditions (given under ‘Environmental variation’)

Organism	Resistance	Type of measurement	Environmental variation	Reference
*Escherichia coli*	Nor	Competition	Mouse/*in vitro*	Lindgren et al. (2005)
	Rif, Str	Competition	Macrophages/laboratory medium	Miskinyte and Gordo (2013)
	Amp, Rif, Str, Tri	Growth rate	Nutrients, salt, pH	Petersen et al. (2009)
	Nal, Rif, Str	Competition	Nutrients, temperature	Trindade et al. (2012)
*Mycobacterium tuberculosis*	Rif	Growth rate	Macrophages/laboratory medium	Mariam et al. (2004)
*Pseudomonas aeruginosa*	Rif	Growth rate	Growth inhibitors	Hall et al. (2011)
*Pseudomonas fluorescens*	Nal	Growth yield	Carbon source	Bataillon et al. (2011)
	Rif	Growth yield	Carbon source	Hall (2013)
*Salmonella enterica* var. Typhimurium	Fus	Competition	Mouse/*in vitro*	Björkman et al. (2000)
	Str	Growth rate	Carbon source	Paulander et al. (2009)
	Rif	Competition	Colony age	Wrande et al. (2008)
*Streptococcus pneumoniae*	Gem	Competition	Mouse nasopharynx/lung/*in vitro*	Johnson et al. (2005)

*Amp*, ampicillin; *Fus*, fusidic acid; *Gem*, gemifloxacin; *Nal*, nalidixic acid; *Nor*, norfloxacin; *Rif*, rifampicin; *Str*, streptomycin; *Tri*, trimethoprim.

**Box 1: Glossary**

*Resistance mechanism* – a physiological process that increases bacterial growth or survival relative to isogenic bacteria lacking the resistance mechanism at concentrations of antibiotics that reduce growth or survival of the latter. Resistance mechanisms include drug efflux, enzymatic modification, and drug-target binding inhibition (Walsh 2000). The proteins involved in resistance mechanisms are frequently encoded on mobile genetic elements including plasmids and integrons or by specific alleles of chromosomal genes.

*Resistance allele* – a variant of a genetic element that results in expression of a resistance mechanism. For example, several alternative mutations in *rpoB* can confer resistance to rifampicin in *E. coli* (Garibyan et al. 2003); the specific nucleotide substitution resulting in increased resistance is the resistance allele, and the resistance mechanism is drug-target binding inhibition (Trinh et al. 2006; Sezonov et al. 2007). If resistance is encoded by an entire genetic element that is absent in sensitive cells, such as a plasmid, then we may consider the presence/absence of the plasmid to be alternative ‘alleles’.

*Trait effect* – change in a phenotypic trait resulting from the presence of a resistance allele. Resistance alleles will frequently affect multiple traits at different levels of organization, and those effects can vary considerably depending on environmental conditions (sections ‘Biological basis of costs of resistance’–‘Environmental variation of trait and selective effects’) and genetic background, including other resistance alleles or compensatory mutations (section ‘Epistatic variation of trait and selective effects’).

*Selective effect*–change in the frequency of a resistance allele in a population over time due to differential survival and reproduction relative to other genotypes, commonly expressed as a selection coefficient (*s*) *in vitro* or competitive index (CI) *in vivo*. That is, a resistance allele with a negative selective effect will decline in frequency relative to genotypes in the same population lacking the resistance allele. Selective effects can be expressed at any concentration of antibiotics. We note that a change in allele frequencies does not always indicate selection; random processes such as genetic drift can also alter allele frequencies.

*Cost of resistance* – synonymous with a negative selective effect in this review. In the literature ‘cost of resistance’ has been used to refer to effects on individual traits such as growth rate or yield (typically estimated experimentally by doubling time during exponential growth and population size at stationary phase, respectively).

**Figure 1:**
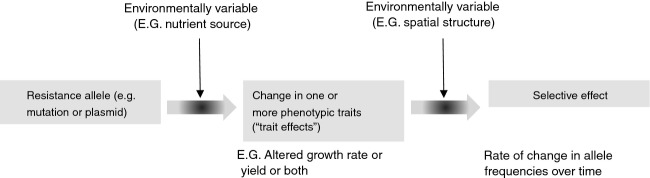
Effects of resistance alleles at the level of individual traits and allele frequencies. The trait effects depicted here, and consequently their selective effects, can also vary depending on genetic factors, such as the presence of compensatory mutations or other resistance alleles.

Here, we ask whether variation in the evolutionary dynamics of antibiotic resistance can be understood by discriminating effects at the level of individual phenotypic traits and at the level of allele frequencies (Fig.1). This approach potentially allows identification of resistance mechanisms or alleles that are consistently under negative selection in different environmental conditions or genetic backgrounds. This is relevant to the management of resistance in pathogenic populations. For example, resistance mechanisms or alleles that are consistently under negative selection in drug-free conditions may be managed by scaling back antibiotic usage, but this will be less effective for resistance mechanisms that are not consistently under negative selection in drug-free conditions. The information required to link resistance evolution in the laboratory to real-world epidemiological dynamics of resistance determinants is increasingly available, partly because DNA sequencing technology now permits the genetic basis of resistance to be identified in individual outbreaks or chronic infections, allowing not only mechanisms but specific alleles to be monitored (Brockhurst et al. 2011; Snitkin et al. 2012; Palmer and Kishony 2013).

## Biological basis of costs of resistance

From the perspective of reducing resistance, the most relevant costs are those that ultimately manifest as a decrease in the frequency of resistant bacteria in a population or metapopulation, that is, negative selective effects. What biological processes are responsible for the changes in phenotypic traits that ultimately lead to such a change in the composition of a population? Two ways how resistance mechanisms can incur such selective effects are quite obvious: they can slow down cellular functions and thus decrease the rate at which bacteria grow and divide, or they can divert building blocks or energy from the growth of biomass, and thus lower yield, that is, the number of bacteria that emerge out of a fixed amount of resources. These two scenarios are not mutually exclusive; some resistance mechanisms may have correlated effects on both rate and yield (Fitzsimmons et al. 2010). These reductions in growth rate or yield or both can then result in a decreased frequency of resistant bacteria in a population. The focus on rate and yield is a simplification, as other biological traits will also influence the dynamics of genotype frequencies. For example, survival during exponential growth or after cessation of growth and entry into stationary phase can differ between genotypes and will translate into selective effects. Reduced survival during exponential growth, for example, will translate into a reduction in the *net* growth rate of a clonal population. A broader definition of growth rate and yield can incorporate contributions of other traits. In section ‘Environmental variation of trait and selective effects’, we come back to the issue of trait effects other than rate and yield that lead to selective effects and discuss how resistance mechanisms can impact gene expression and regulatory responses to stressful conditions.

**Figure 2:**
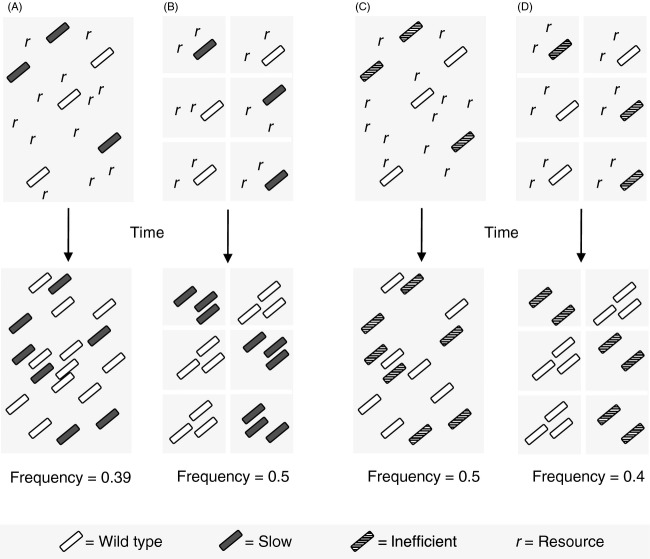
Selective effects of alleles, such as antibiotic-resistance alleles in the absence of drugs, that reduce growth rate (‘slow’ – A, B) or yield (‘inefficient’ – C, D) in unstructured (A, C) and structured (B, D) populations. The wild type converts one unit of resource *r* to produce one additional cell, and growth continues, with no cell death, until all resources are depleted. ‘Slow’ bacteria produce new cells at a lower rate than the wild type but with the same efficiency, causing them to decline in frequency in spatially unstructured conditions where resources are shared. ‘Inefficient’ bacteria produce new cells at the same rate but use 2 × *r* per new cell, declining in frequency across a spatially structured metapopulation where clonal demes consume resources ‘privately’ (Pfeiffer et al. 2001; Bachmann et al. 2013).

These considerations make it evident that costs of resistance are inherently context-dependent. This is for two reasons. First, whether a change in a cellular function impacts growth rate or yield depends on external conditions. In some conditions, a given function might limit the rate at which bacteria grow, or the yield they achieve. In other conditions, the same function might not limit rate or yield, and a reduction in this function due to the presence of an antibiotic resistance allele will thus not affect these two traits. Second, whether a reduction in growth rate or yield impact the frequency of the resistant type over time depends on the population structure and whether growth resources are shared or private (Pfeiffer et al. 2001; MacLean and Gudelj 2006; Frank 2010, 2014). In well-mixed environments with shared resources, genotype frequencies over time are mostly dependent on growth rate. In other conditions, for example, when clonal populations inhabit patches with limited dispersal, genotype frequencies, expressed as a fraction of the total metapopulation, can depend mostly on yield (Fig.2). This has been demonstrated experimentally in populations of *Lactococcus lactis* (Bachmann et al. 2013). This study, like Fig.2, assumed no migration among patches. In reality, spatially structured populations will often be subject to some degree of migration and genetic mixing. Theory suggests that as mixing increases, greater resource competition shifts the balance toward high-rate, rather than high-yield, phenotypes (Pfeiffer et al. 2001; Frank 2010). Based on these considerations, we would predict changes in the costs of resistance across external conditions to be the norm rather than the exception.

To explain observed variation in costs of resistance, we suggest breaking the problem of identifying costs in two parts. The first part describes how a given resistance mechanism affects the rate at which bacteria grow or the yield they achieve. Because changes in rate and yield may be determined by trait effects at other levels of organization (section ‘Environmental variation of trait and selective effects’), such inference can be based on molecular microbiological information (Ruusala et al. 1984; Brandis et al. 2012), metabolic control analysis (Dykhuizen and Dean 1990; Fell and Cornish-Bowden 1997), or related approaches that describe how the growth characteristics of an organism depend on external factors such as availability of limiting nutrients (Mortlock 1984; Lendenmann et al. 1996). The second part is then to analyze how trait effects influence the frequency and total abundance of the resistant type in a population or metapopulation. Note that in certain conditions changes in rate or yield may alter population-level growth without affecting allele frequencies, such as a reduced yield in spatially nonstructured conditions (Fig.2C). This analysis can be based on a well-developed body of theory on how the action of natural selection depends on population structure (Levin 1976; Caswell 1989; Charlesworth 1994; Hanski 1999).

To make these considerations more concrete, consider the example of a bacterium with a gene encoding a *β*-lactamase conferring resistance to penicillins. What are the ‘costs’ of this resistance mechanism? Let us first focus on trait effects. An important distinction is whether the *β*-lactamase is constitutively expressed or whether expression is induced by the presence of antibiotics (Minami et al. 1980; Livermore 1995). In the latter case, bacteria that grow in the absence of antibiotics will produce none, or only very little, of the *β*-lactamase. The presence of the gene itself might still have cellular consequences that could manifest as marginal costs. Under conditions where the doubling time of the bacterium is limited by the time needed to replicate the chromosome, the presence of an extra gene on the chromosome will increase the time, although only slightly so (if the replication time is proportional to the length of the chromosome, then the increase will be about 0.1% per additional gene for a bacterium with a chromosome of 1000 genes, and even smaller for bacteria with more genes). Likewise, the extra building blocks required for the increased size of the chromosome are expected to be marginal. The resources required for DNA are a small fraction of a cell's total resources, and an increase in DNA content resulting from the addition of one gene thus has small consequences (Brinas et al. 2003).

The main trait effects in drug-free conditions are therefore expected to result from expression of the gene and production of the *β*-lactamase (Dekel and Alon 2005; Stoebel et al. 2008). What are these effects? The production of a protein requires building blocks assimilated from carbon, nitrogen, and energy. If any of these three resources are limiting the growth rate or yield of bacteria, in that an increase or decrease in the resource causes a concordant increase or decrease in rate or yield, then expressing the *β*-lactamase will reduce the trait in question, and the magnitude of this reduction will depend on the amount of resistance protein produced relative to the total biomass of a cell. Alternatively, it is conceivable (and realistic for many natural situations) that growth rate and yield are limited by other elements, for example iron or sulfur (Joyner and Lindow 2000; Mann and Chisholm 2000; Gourion et al. 2006). In these cases, one would expect that investing carbon, nitrogen, and energy into producing *β*-lactamases would have virtually no consequences for a bacterium's growth traits, with the caveat that investing these resources into antibiotic resistance could lead to reduced investment into the machinery for the acquisition of iron and sulfur, or expression of the protein itself may pleiotropically affect other traits, and thus indirectly reduce the growth rate or yield.

Let us now consider conditions where the growth yield of the bacterium is limited by nitrogen, and where the production of large amounts of *β*-lactamase diverts nitrogen away from the production of cellular biomass, reducing the number of bacteria that can be formed from a given amount of resources, that is, the yield. How this trait effect translates into a selective effect, that is, how it influences the frequency of the resistant type, depends on the population structure. Specifically, if resistant and sensitive bacteria compete in a well-mixed environment, then the reduced yield of the resistant bacterium is not expected to result in a competitive disadvantage (Pfeiffer et al. 2001; Frank 2010). The reduction in available nitrogen resulting from the production of *β*-lactamase affects both types equally and does not lead to a competitive imbalance. By contrast, if resources occur in patches that are colonized by one or a few individuals that expand into clonal populations before they disperse again, then the yield that a clone achieves can be decisive for its competitive success (Fig.2). We will revisit context-dependent costs below and discuss how these considerations help explain previously observed variation in the measured costs of resistance.

## Environmental variation of trait and selective effects

Consistent with the above rationale that trait and selective effects are context-dependent, several independent experiments with different types of antibiotic-resistant bacteria show that the phenotypic effects of resistance alleles and their influence on allele frequencies over time vary strongly depending on environment (Table 1). In these studies, trait effects such as changes in growth rate or yield and selective effects as inferred by competition assays are both referred to as ‘costs of resistance’. We suggest that, particularly given environmental variation of these effects, distinguishing trait and selective effects is beneficial.

For example, many *in vitro* investigations of ‘costs’ in different conditions are based on growth rate measurements (Nagaev et al. 2001; Nilsson et al. 2006; MacLean and Buckling 2009; Paulander et al. 2009; Petersen et al. 2009; Hall et al. 2011). They are usually performed in well-mixed conditions in liquid culture, where a reduction in growth rate is likely to translate to a negative selective effect. That is, in these conditions, the trait effect (change in growth rate) gives a reliable indication of the likely selective effect of resistance, so the failure to distinguish the two is not critical. Consistent with this, some studies have demonstrated a positive association between effects of resistance mechanisms in growth rate assays and pairwise competitions (Petersen et al. 2009; Perron et al. 2010; Guo et al. 2012). However, if the same type of experiments were carried out in spatially structured environments, selective effects may be more sensitive to changes in yield than growth rate (Kreft 2004; Bachmann et al. 2013). In such conditions, changes in maximum growth rate are no longer a reliable indicator of selective effects, and discriminating the two types of effect is more important for understanding the incidence of resistance over time. Crucially, growth in spatially structured environments with distinct subpopulations is probably more realistic than growth in well-mixed environments for many pathogenic species. Therefore, if we aim to investigate costs of resistance across realistic settings, it is potentially misleading to group both types of effect under the same term, given that the same trait effect may translate to different selective effects in different environments.

The relevance of this problem is demonstrated by comparing selective effects estimated for the same genotypes *in vitro* and *in vivo*. Several experimental studies (Björkman et al. 1998; Nilsson et al. 2004; Marcusson et al. 2009), as well as a meta-analysis (T. Vogwill and R. C. MacLean, unpublished data), indicate a positive overall association: Resistance alleles that are under negative selection *in vitro* tend to also be under negative selection *in vivo*. However, in other studies, the association is weaker, and the rank order of different resistance alleles in terms of their selective effects can differ between assay conditions (Table 1). A key factor appears to be the mechanism of antibiotic resistance. For example, data presented by Björkman et al. (2000) show a strong positive correlation between *in vitro* and *in vivo* selective effects for various streptomycin-resistance alleles (*r*^2^ = 0.56, *P* = 0.02), but no significant correlation for fusidic acid-resistance alleles (*r*^2^ = 0.13, *P* = 0.13). The lack of association here could be due to variation of the trait effects of individual alleles across assay conditions, or because the same trait effects result in different selective effects *in vitro* and *in vivo*. For instance, a mouse is more spatially structured compared to a shaken test tube or microplate, and therefore, selective effects *in vivo* may be more sensitive to changes in yield than rate. We note that *in vivo* and *in vitro* conditions will also differ in other ways such as nutrient availability and interaction with the host immune system, which could also modify trait and selective effects. In such scenarios, our understanding of variation of selective effects across conditions may be improved by examining the effects of specific resistance alleles on individual traits like growth rate and yield, and testing their correlation with selective effects observed in competition experiments.

We have so far focused on trait effects at the level of growth parameters such as rate and yield, because these are the most frequently measured and are the ultimate basis of selective effects. However, the bacterial phenotype is composed of a vast number of traits. Resistance alleles may have multiple trait effects at different levels of organization, and those trait effects may vary environmentally. For example, streptomycin-resistance mutations on *rpsL* directly impair translation, which reduces growth rate and has a negative selective effect in rich growth media (Kurland 1992). However, the same mutations repress induction of a stress-associated *σ*-factor in minimal media containing poor carbon sources, resulting in a dampened stress response and rapid growth compared to the wild type (Paulander et al. 2009). Altered stress responses are also implicated in the increased survival of streptomycin-resistant mutants inside macrophages (Miskinyte and Gordo 2013). Thus, a predictive understanding of the effects of resistance alleles on bacterial growth parameters may be gained by quantifying their effects on specific processes, in this case translation and stress response induction, in different conditions.

Resistance alleles that pleiotropically affect multiple traits are probably very common. Mutations that confer antibiotic resistance by modifying the cellular target of an antibiotic typically occur on genes encoding proteins involved in essential functions for cellular growth and survival (Walsh 2000). As in the example of *rpsL* mutations above, such mutations can have wide-ranging consequences for the expression of other genes or the activity of pathways that are not directly related to the function of the mutated enzyme. If the influence of these pleiotropic effects on growth rate or yield varies environmentally, this may result in variation of the selective effects of resistance alleles across environments, even if the relationship between rate or yield and selective effects is constant. This is illustrated by the observation that rifampicin-resistance mutations on RNA polymerase can influence expression across the entire genome (Applebee et al. 2008; Conrad et al. 2010; Derewacz et al. 2013) and pleiotropically influence other traits that are not directly linked to transcription, such as metabolism of carbon sources (Jin and Gross 1989; Perkins and Nicholson 2008; Paulander et al. 2009). As a result, rifampicin-resistance mutations can be positively selected in some antibiotic-free conditions, such as adaptation to novel carbon sources (Applebee et al. 2008; Conrad et al. 2010; Tenaillon et al. 2012), aging colonies (Wrande et al. 2008), or high temperature (Rodríguez-Verdugo et al. 2013).

Alleles that confer resistance via efflux pumps also tend to have pleiotropic effects, because they often have broad activities against antibiotics and other compounds (Nikaido 1998; Piddock 2006; Nikaido and Pagès 2012). This is relevant for the distribution of resistance in pathogenic populations because a single allele encoding resistance against multiple antibiotics permits coselection, where positive selection for resistance to one drug causes resistance to other drugs to spread. Indeed, coselection has been implicated in the persistence of some resistance mechanisms, such as trimethoprim resistance in *Escherichia coli* in Sweden (Sundqvist et al. 2010) and sulfonamide resistance in *E. coli* in the United Kingdom (Enne et al. 2001; Bean et al. 2005), despite restrictions on use of these antibiotics. Coselection of resistance alleles may also result from selection for resistance to heavy metals present as contaminants in the environment (Baker-Austin et al. 2006). In scenarios where coselection is possible, such as populations that are exposed to different drugs over time, the selective effects of resistance alleles across conditions are best understood by quantifying trait effects in terms of resistance to multiple compounds. Recently, this approach has been used to predict the effects of different interventions by screening for cross-resistance and collateral sensitivity among clinically relevant combinations of drugs (D'Costa et al. 2006; Imamovic and Sommer 2013; Lázár et al. 2013).

Coselection can also occur when multiple resistance alleles occur on the same genetic background. For example, plasmids and resistance gene cassettes often encode resistance against multiple antibiotics on separate genes (Alekshun and Levy 2007; Chambers and Deleo 2009; San Millan et al. 2014). Multidrug resistance can also be acquired via sequential acquisition of different resistance mutations on chromosomal genes (Livermore 2002; Da Silva and Palomino 2011). Interestingly, in cases where multiple resistance alleles are present on the same genome, plasmid, or lineage of cells, their net trait effects and resultant selective effects in combination may deviate from what we would predict based on their independent effects. Therefore, understanding the potential for coselection requires that we consider epistatic variation of trait and selective effects. More generally, variation of trait or selective effects depending on genetic background (epistasis) constrains our ability to translate *in vitro* results to real-world scenarios, because evolving pathogenic populations will differ genetically from laboratory strains. We next discuss whether this type of variation can also be understood by distinguishing trait effects and selective effects of resistance alleles.

## Epistatic variation of trait and selective effects

Recent work shows that the trait effects of resistance alleles, expressed as changes in growth rate or yield in the absence of antibiotics (Ward et al. 2009; Hall and MacLean 2011) or at inhibitory antibiotic concentrations (Weinreich et al. 2006; Salverda et al. 2011), vary strongly depending on the presence of other resistance alleles on the same genetic background. Furthermore, studies that have measured selective effects *in vitro* through competition assays in the absence of antibiotics have revealed pervasive epistasis between resistance alleles (Rozen et al. 2007; Trindade et al. 2009; Silva et al. 2011). That is, the same resistance allele may have different selective effects across genetic backgrounds that vary at other loci involved in antibiotic resistance. These interactions are relevant for understanding resistance in pathogenic populations. For example, recent work shows that combinations of mutations associated with a small or no negative selective effect in the absence of antibiotics *in vitro* appear to be overrepresented among clinical isolates of *Mycobacterium tuberculosis* (Borrell et al. 2013), indicating that laboratory measurements can be predictive of evolutionary dynamics in natural hosts. In some scenarios, the net selective effect of multiple resistance alleles may be understood by considering how the trait effects of one allele can be influenced by the presence of another resistance allele.

For example, the negative selective effects in drug-free conditions of rifampicin-resistance mutations on *rpoB* tend to be buffered by the presence of streptomycin-resistance mutations on *rpsL* (Trindade et al. 2009). Independently, these resistance mechanisms impair transcription and translation, respectively (Kurland 1992; Reynolds 2000), and this is associated with reduced growth rate. However, molecular studies show that activity of these enzymes is very closely related (Dutta et al. 2011), and impairment of one can indirectly inhibit the other (Proshkin et al. 2010), effectively placing a speed limit on the transcription-translation pathway. Thus, the influence of an *rpoB* mutation on transcription and bacterial growth rate may be relatively small in conditions where ribosomal activity is inhibited. Consistent with this, the negative effects of *rpoB* mutations on bacterial growth rate can be reduced by the addition of ribosome inhibitors (Hall et al. 2011). In such conditions, reduced growth rates typically translate to negative selective effects (T. Vogwill and R. C. MacLean, unpublished data), so the prevalence of antagonistic epistasis between *rpoB* and *rpsL* mutations in their selective effects (Trindade et al. 2009) may be explained by antagonism between their effects on growth rate (Ward et al. 2009). It is not yet known whether their effects on transcription and translation also interact epistatically. Nevertheless, combining molecular microbiological information with measurement of trait and selective effects could also be applied to other combinations of resistance mechanisms, because the molecular basis of resistance is known for many combinations of bacteria and drugs.

Epistatic interactions have also been observed among resistance mutations on the same gene. For example, in a *β*-lactamase, epistatic interactions in terms of mutational effects on resistance against cefotaxime have been explained by examining interactive changes in enzyme activity and thermodynamic stability (Salverda et al. 2011; Schenk et al. 2014) (see also Bershtein et al. (2006)). Given that almost all mutations affect stability (DePristo et al. 2005; Tokuriki et al. 2007) and that stability is a key determinant of the effective concentration of functional enzymes in a cell (Pakula and Sauer 1989), the same approach could be applied to understand the physiological basis of trait effects in the absence of antibiotics for resistance mechanisms that involve multiple mutations on the same chromosomal gene, such as fluoroquinolone resistance in *E. coli* (Lindgren et al. 2003, 2005; Marcusson et al. 2009). For detailed reviews of the mechanistic drivers of epistasis see Lehner (2011) or de Visser et al. (2011).

Epistasis at the level of trait and selective effects may also influence the distribution of antibiotic resistance in scenarios where a resistance allele modulates the effects of mutations at other sites that are not directly involved in resistance mechanisms, such as compensatory mutations that have a positive selective effect only for genotypes with resistance alleles. Compensatory mutations can modify the effect of various resistance mechanisms [reviewed by Maisnier-Patin and Andersson (2004) both *in vitro* (Schrag et al. 1997; Maisnier-Patin et al. 2002; Brandis et al. 2012) and *in vivo* (Björkman et al. 2000)]. There is mounting evidence that compensatory mutations are not only quickly selected in laboratory settings, but are common in resistant clinical isolates (Shcherbakov et al. 2010; Comas et al. 2012; de Vos et al. 2013). Therefore, even in cases where the effects of a resistance allele on growth traits are understood, their influence on the effects of mutations at other loci should also be considered.

The trait effects of alleles at other loci can also help to identify the trait effects of the original resistance allele that are under negative selection in the absence of antibiotics, that is, the physiological basis of costs of resistance. For example, expression of some resistance genes is induced by the antibiotic itself (Minami et al. 1980; Depardieu et al. 2007; Foucault et al. 2010). The fact that such regulatory mechanisms have evolved indicates that spurious expression (transcription and translation) of resistance genes in antibiotic-free conditions has a negative selective effect. Consistent with this, induction of expression of *tetA* in the absence of tetracycline incurs high costs in competition experiments in *E. coli* K12 (Nguyen et al. 1989), while without induction, the resistance allele has no measurable selective effect. Similarly, vancomycin resistance has a negative selective effect when expression is induced in the absence of antibiotics both *in vivo* and *in vitro* (Foucault et al. 2010). Therefore, analysis of the trait effects of alleles at other loci, including regulatory mechanisms and compensatory mutations, may provide insight into the biological basis of the cost of the original resistance allele.

## Conclusions and future directions

The take-home message of this review is that variation of ‘costs of resistance’ can be better understood by distinguishing effects on individual traits and on genotype frequencies over time. It is unnecessarily misleading to group changes in growth rate or yield in pure culture and selective effects inferred from *in vitro* or *in vivo* competitions all under the same term. The motivation and justification for investigating ‘costs of resistance’ are typically to gain insight into the likelihood that resistance will persist or decline in the absence of antibiotics. In a given environment, this is defined by selective effects as inferred from changes in genotype frequencies, rather than trait effects such as changes in growth rate or yield. For many types of resistance and environmental conditions, measured trait effects such as growth rate are strong predictors of selective effects, but this is not always the case. In some cases, resistance alleles may have important trait effects that do not result in a selective effect, such as reduced yield in spatially nonstructured conditions (Fig.2C). Therefore, we do not suggest that trait effects are unimportant or that selective effects are the only relevant parameter for managing resistant infections, only that disentangling the effects of resistance alleles at different levels of organization will be beneficial and that using more specific terminology would be a good start.

Such information can potentially be applied to the management of resistance. For example, when the physiological basis of selective effects is understood, novel strategies may be devised to maximize costs by environmental manipulation, ultimately accelerating the decline of resistance. In the case of resistance genes associated with regulatory mechanisms, one way to achieve this would be via compounds that induce costly expression of resistance genes in the absence of antibiotics, such as analogues of relevant drugs (Nguyen et al. 1989). A similar approach is plausible for resistance alleles on chromosomal genes, such as streptomycin-resistance mutations on *rpsL* or rifampicin-resistance mutations on *rpoB*. In these examples, the knowledge that selective effects are often related to defective translation or transcription (Kurland 1992; Reynolds 2000) suggests that conditions where the rate of gene expression limits growth rate, and selective effects are correlated with growth rate effects, are less likely to sustain resistance in the absence of antibiotics. Such conditions might be artificially created using signaling molecules that induce expression of the many quorum-sensing-regulated genes, typically acyl-homoserine lactones for Gram-negative and processed oligo-peptides for Gram-positive bacteria (Whiteley et al. 1999; Miller and Bassler 2001; Hall et al. 2011), thereby increasing the total number of genes expressed and potentially increasing the likelihood that impaired transcription or translation because the presence of resistance alleles reduces bacterial growth rate. As a first step, these and other interventions aimed at increasing costs of particular resistance alleles could be investigated *in vitro*.

More generally, understanding whether costs are consistently low or high for a given type of resistance across alternative alleles and environments will help to predict whether restricted usage of drugs will work. We suggest a pluralist approach to this problem, with at least three valuable types of information. First, molecular microbiological information allows relevant trait effects to be identified, as in the example of *rpsL* mutations that affect both translation and stress response induction (Paulander et al. 2009). Second, comparing the selective effect of the same resistance allele across different conditions, both *in vitro* and *in vivo*, indicates whether trait and selective effects identified in one environment translate to other conditions. Continuing with the example of *rpsL* mutations, the role of altered stress response induction as observed *in vitro* was translated to a clinically relevant environment (macrophages) by Miskinyte and Gordo (2013). In general, the influence of population structure on selective effects of resistance alleles is particularly important given that many pathogenic species grow in spatially structured biofilms (Costerton et al. 1995; Kreft 2004). Third, in an epidemiological context, we are concerned not only with selective effects within a given host, but in a population of hosts that are connected, from the pathogen's perspective, by transmission. Therefore, comparing selective effects *in vitro* and *in vivo* to the distribution of the same resistance alleles in collections of clinical isolates provides a key test of how informative experimental evolutionary dynamics are in real pathogenic populations. This approach was recently applied successfully in *Mycobacterium smegmatis* and *tuberculosis* by Borrell et al. (2013).

## References

[b1] Alekshun MN, Levy SB (2007). Molecular mechanisms of antibacterial multidrug resistance. Cell.

[b2] Andersson DI (2003). Persistence of antibiotic resistant bacteria. Current Opinion in Microbiology.

[b4] Andersson DI, Hughes D (2010). Antibiotic resistance and its cost: is it possible to reverse resistance?. Nature Reviews Microbiology.

[b3] Andersson DI, Hughes D (2011). Persistence of antibiotic resistance in bacterial populations. FEMS Microbiology Reviews.

[b5] Andersson DI, Levin BR (1999). The biological cost of antibiotic resistance. Current Opinion in Microbiology.

[b6] Applebee MK, Herrgård MJ, Palsson BO (2008). Impact of individual mutations on increased fitness in adaptively evolved strains of *Escherichia coli*. Journal of Bacteriology.

[b7] Arason VA, Gunnlaugsson A, Sigurdsson JA, Erlendsdottir H, Gudmundsson S, Kristinsson KG (2002). Clonal spread of resistant *Pneumococci* despite diminished antimicrobial use. Microbial Drug Resistance.

[b8] Bachmann H, Fischlechner M, Rabbers I, Barfa N, Branco dos Santos F, Molenaar D, Teusink B (2013). Availability of public goods shapes the evolution of competing metabolic strategies. Proceedings of the National Academy of Sciences of the United States of America.

[b9] Baker-Austin C, Wright MS, Stepanauskas R, McArthur JV (2006). Co-selection of antibiotic and metal resistance. Trends in Microbiology.

[b10] Baquero F, Negri MC (1997). Selective compartments for resistant microorganisms in antibiotic gradients. BioEssays.

[b11] Bataillon T, Zhang T, Kassen R (2011). Cost of adaptation and fitness effects of beneficial mutations in *Pseudomonas fluorescens*. Genetics.

[b12] Bean DC, Livermore DM, Papa I, Hall LM (2005). Resistance among *Escherichia coli* to sulphonamides and other antimicrobials now little used in man. Journal of Antimicrobial Chemotherapy.

[b13] Bergstrom CT, Feldgarden M, Stearns SC, Koella JC (2008). The ecology and evolution of antibiotic-resistant bacteria. Evolution in Health and Disease.

[b14] Bershtein S, Segal M, Bekerman R, Tokuriki N, Tawfik DS (2006). Robustness-epistasis link shapes the fitness landscape of a randomly drifting protein. Nature.

[b15] Björkman J, Hughes D, Andersson DI (1998). Virulence of antibiotic-resistant *Salmonella typhimurium*. Proceedings of the National Academy of Sciences of the United States of America.

[b16] Björkman J, Nagaev I, Berg OG, Hughes D, Andersson DI (2000). Effects of environment on compensatory mutations to ameliorate costs of antibiotic resistance. Science.

[b17] Borrell S, Teo Y, Giardina F, Streicher EM, Klopper M, Feldmann J, Müller B (2013). Epistasis between antibiotic resistance mutations drives the evolution of extensively drug-resistant tuberculosis. Evolution, Medicine, and Public Health.

[b18] Brandis G, Wrande M, Liljas L, Hughes D (2012). Fitness-compensatory mutations in rifampicin-resistant RNA polymerase. Molecular Microbiology.

[b19] Brinas L, Moren MA, Teshager T, Zarazaga M, Saenz Y, Porrero C, Dominguez L (2003). β-lactamase characterization in *Escherichia coli* isolates with diminished susceptibility or resistance to extended-spectrum cephalosporins recovered from sick animals in Spain. Microbial Drug Resistance.

[b20] Brockhurst MA, Colegrave N, Rozen DE (2011). Next-generation sequencing as a tool to study microbial evolution. Molecular Ecology.

[b21] Caswell H (1989). Matrix Population Models.

[b22] Chambers HF, Deleo FR (2009). Waves of resistance: *Staphylococcus aureus* in the antibiotic era. Nature Reviews Microbiology.

[b23] Charlesworth B (1994). Evolution in Age-Structured Populations.

[b24] Chevin LM (2011). On measuring selection in experimental evolution. Biology Letters.

[b25] Cohen T, Sommers B, Murray M (2003). The effect of drug resistance on the fitness of *Mycobacterium tuberculosis*. The Lancet.

[b26] Comas I, Borrell S, Roetzer A, Rose G, Malla B, Kato-Maeda M, Galagan J (2012). Whole-genome sequencing of rifampicin-resistant *Mycobacterium tuberculosis* strains identifies compensatory mutations in RNA polymerase genes. Nature Genetics.

[b27] Conrad TM, Frazier M, Joyce AR, Cho BK, Knight EM, Lewis NE, Landick R (2010). RNA polymerase mutants found through adaptive evolution reprogram *Escherichia coli* for optimal growth in minimal media. Proceedings of the National Academy of Sciences of the United States of America.

[b28] Costerton JW, Lewandowski Z, Caldwell DE, Korber DR, Lappin-Scott HM (1995). Microbial biofilms. Annual Review of Microbiology.

[b29] Da Silva PEA, Palomino JC (2011). Molecular basis and mechanisms of drug resistance in *Mycobacterium tuberculosis*: classical and new drugs. Journal of Antimicrobial Chemotherapy.

[b30] D'Costa VM, McGrann KM, Hughes DW, Wright GD (2006). Sampling the antibiotic resistome. Science.

[b31] Dekel E, Alon U (2005). Optimality and evolutionary tuning of the expression level of a protein. Nature.

[b32] Depardieu F, Podglajen I, Leclercq R, Collatz E, Courvalin P (2007). Modes and modulations of antibiotic resistance gene expression. Clinical Microbiology Reviews.

[b33] DePristo MA, Weinreich DM, Hartl DL (2005). Missense meanderings in sequence space: a biophysical view of protein evolution. Nature Reviews Genetics.

[b34] Derewacz DK, Goodwin CR, McNees CR, McLean JA, Bachmann BO (2013). Antimicrobial drug resistance affects broad changes in metabolomic phenotype in addition to secondary metabolism. Proceedings of the National Academy of Sciences of the United States of America.

[b35] Dutta D, Shatalin K, Epshtein V, Gottesman ME, Nudler E (2011). Linking RNA polymerase backtracking to genome instability in *E. coli*. Cell.

[b36] Dykhuizen DE, Dean AM (1990). Enzyme activity and fitness – evolution in solution. Trends in Ecology & Evolution.

[b37] Enne VI, Livermore DM, Stephens P, Hall LM (2001). Persistence of sulphonamide resistance in *Escherichia coli* in the UK despite national prescribing restriction. Lancet.

[b38] Fell D, Cornish-Bowden A (1997). Understanding the Control of Metabolism.

[b39] Fitzsimmons JM, Schoustra SE, Kerr JT, Kassen R (2010). Population consequences of mutational events: effects of antibiotic resistance on the *r**K* trade-off. Evolutionary Ecology.

[b40] Foucault ML, Depardieu F, Courvalin P, Grillot-Courvalin C (2010). Inducible expression eliminates the fitness cost of vancomycin resistance in enterococci. Proceedings of the National Academy of Sciences of the United States of America.

[b41] Frank SA (2010). The trade-off between rate and yield in the design of microbial metabolism. Journal of Evolutionary Biology.

[b42] Frank SA (2014). Microbial metabolism: optimal control of uptake versus synthesis. PeerJ.

[b43] Garibyan L, Huang T, Kim M, Wolff E, Nguyen A, Nguyen T, Diep A (2003). Use of the *rpoB* gene to determine the specificity of base substitution mutations on the *Escherichia coli* chromosome. DNA Repair.

[b44] Gottesman BS, Carmeli Y, Shitrit P, Chowers M (2009). Impact of quinolone restriction on resistance patterns of *Escherichia coli* isolated from urine by culture in a community setting. Clinical Infectious Diseases.

[b45] Gourion B, Rossignol M, Vorholt JA (2006). A proteomic study of *Methylobacterium extorquens* reveals a response regulator essential for epiphytic growth. Proceedings of the National Academy of Sciences of the United States of America.

[b46] Gullberg E, Cao S, Berg OG, Ilbäck C, Sandegren L, Hughes D, Andersson DI (2011). Selection of resistant bacteria at very low antibiotic concentrations. PLoS Pathogens.

[b47] Guo BN, Abdelraouf K, Ledesma KR, Nikolaou M, Tam VH (2012). Predicting bacterial fitness cost associated with drug resistance. Journal of Antimicrobial Chemotherapy.

[b48] Hall AR (2013). Genotype-by-environment interactions due to adaptation and antibiotic resistance in *Escherichia coli*. Journal of Evolutionary Biology.

[b49] Hall AR, MacLean RC (2011). Epistasis buffers the fitness effects of rifampicin-resistance mutations in *Pseudomonas aeruginosa*. Evolution.

[b50] Hall AR, Griffiths VF, MacLean RC, Colegrave N (2010). Mutational neighbourhood and mutation supply rate constrain adaptation in *Pseudomonas aeruginosa*. Proceedings of the Royal Society B: Biological Sciences.

[b51] Hall AR, Iles JC, MacLean RC (2011). The fitness cost of rifampicin resistance in *Pseudomonas aeruginosa* depends on demand for RNA polymerase. Genetics.

[b52] Hanski I (1999). Metapopulation Ecology.

[b53] Hermsen R, Deris JB, Hwa T (2012). On the rapidity of antibiotic resistance evolution facilitated by a concentration gradient. Proceedings of the National Academy of Sciences of the United States of America.

[b54] Imamovic L, Sommer MO (2013). Use of collateral sensitivity networks to design drug cycling protocols that avoid resistance development. Science Translational Medicine.

[b55] Jin DJ, Gross CA (1989). Characterization of the pleiotropic phenotypes of rifampin-resistant *rpoB* mutants of *Escherichia coli*. Journal of Bacteriology.

[b56] Johnson CN, Briles DE, Hollingshead WH, Benjamin SK, Waites KB (2005). Relative fitness of fluoroquinolone-resistant *Streptococcus pneumoniae*. Emerging Infectious Diseases.

[b57] Joyner DC, Lindow SE (2000). Heterogeneity of iron bioavailability on plants assessed with a whole-cell GFP-based bacterial biosensor. Microbiology.

[b58] Kim SH, Wei CI (2007). Antibiotic resistance and Caco-2 cell invasion of *Pseudomonas aeruginosa* isolates from farm environments and retail products. International Journal of Food Microbiology.

[b59] Kreft JU (2004). Biofilms promote altruism. Microbiology.

[b60] Kurland CG (1992). Translational accuracy and the fitness of bacteria. Annual Review of Genetics.

[b61] Lázár V, Pal Singh G, Spohn R, Nagy I, Horváth B, Hrtyan M, Busa-Fekete R (2013). Bacterial evolution of antibiotic hypersensitivity. Molecular Systems Biology.

[b62] Lehner B (2011). Molecular mechanisms of epistasis within and between genes. Trends in Genetics.

[b63] Lendenmann U, Snozzi M, Egli T (1996). Kinetics of the simultaneous utilization of sugar mixtures by *Escherichia coli* in continuous culture. Applied and Environmental Microbiology.

[b64] Lenski RE, Rose MR, Simpson SC, Tadler SC (1991). Long-term experimental evolution in *Escherichia coli* 1. Adaptation and divergence during 2,000 generations. American Naturalist.

[b65] Levin SA (1976). Population dynamic models in heterogeneous environments. Annual Review of Ecology and Systematics.

[b66] Levin BR (2001). Minimizing potential resistance: a population dynamics view. Clinical Infectious Diseases.

[b67] Levin BR, Perrot V, Walker N (2000). Compensatory mutations, antibiotic resistance and the population genetics of adaptive evolution in bacteria. Genetics.

[b68] Levy SB, Marshall B (2004). Antibacterial resistance worldwide: causes, challenges and responses. Nature Medicine.

[b69] Lindgren PK, Karlsson A, Hughes D (2003). Mutation rate and evolution of fluoroquinolone resistance in *Escherichia coli* isolates from patients with urinary tract infections. Antimicrobial Agents and Chemotherapy.

[b70] Lindgren PK, Marcusson LL, Sandvang D, Frimodt-Møller N, Hughes D (2005). Biological cost of single and multiple norfloxacin resistance mutations in *Escherichia coli* implicated in urinary tract infections. Antimicrobial Agents and Chemotherapy.

[b71] Livermore DM (1995). β-lactamases in laboratory and clinical resistance. Clinical Microbiology Reviews.

[b72] Livermore DM (2002). Multiple mechanisms of antimicrobial resistance in *Pseudomonas aeruginosa*: our worst nightmare?. Clinical Infectious Diseases.

[b73] MacLean RC, Buckling A (2009). The distribution of fitness effects of beneficial mutations in *Pseudomonas aeruginosa*. PLoS Genetics.

[b74] MacLean RC, Gudelj I (2006). Resource competition and social conflict in experimental populations of yeast. Nature.

[b75] Maisnier-Patin S, Andersson DI (2004). Adaptation to the deleterious effects of antimicrobial drug resistance mutations by compensatory evolution. Research in Microbiology.

[b76] Maisnier-Patin S, Berg OG, Liljas L, Andersson DI (2002). Compensatory adaptation to the deleterious effect of antibiotic resistance in *Salmonella typhimurium*. Molecular Microbiology.

[b77] Maisnier-Patin S, Paulander W, Pennhag A, Andersson DI (2007). Compensatory evolution reveals functional interactions between ribosomal proteins S12 L14 and L19. Journal of Molecular Biology.

[b78] Mann EL, Chisholm SW (2000). Iron limits the cell division rate of *Prochlorococcus* in the eastern equatorial Pacific. Limnology and Oceanography.

[b79] Marcusson LL, Frimodt-Møller N, Hughes D (2009). Interplay in the selection of fluoroquinolone resistance and bacterial fitness. Plos Pathogens.

[b80] Mariam DH, Mengistu Y, Hoffner SE, Andersson DI (2004). Effect of *rpoB* mutations conferring rifampin resistance on fitness of *Mycobacterium tuberculosis*. Antimicrobial Agents and Chemotherapy.

[b81] Martínez JL, Rojo F (2011). Metabolic regulation of antibiotic resistance. Fems Microbiology Reviews.

[b82] Miller MB, Bassler BL (2001). Quorum sensing in bacteria. Annual Review of Microbiology.

[b83] Minami S, Yotsuji A, Inoue M, Mitsuhashi S (1980). Induction of beta-lactamase by various beta-lactam antibiotics in *Enterobacter cloacae*. Antimicrobial Agents and Chemotherapy.

[b84] Miskinyte M, Gordo I (2013). Increased survival of antibiotic-resistant *Escherichia coli* inside macrophages. Antimicrobial Agents and Chemotherapy.

[b85] Mortlock RP (1984). Microorganisms as Model Systems for Studying Evolution.

[b86] Nagaev I, Björkman J, Andersson DI, Hughes D (2001). Biological cost and compensatory evolution in fusidic acid-resistant *Staphylococcus aureus*. Molecular Microbiology.

[b87] Nguyen TNM, Phan QG, Duong LP, Bertrand KP, Lenski RE (1989). Effects of carriage and expression of the Tn10 tetracycline-resistance operon on the fitness of *Escherichia coli* K12. Molecular Biology and Evolution.

[b88] Nilsson AI, Kugelberg E, Berg OG, Andersson DI (2004). Experimental adaptation of *Salmonella typhimurium* to mice. Genetics.

[b89] Nikaido H (1998). Multiple antibiotic resistance and efflux. Current Opinion in Microbiology.

[b90] Nikaido H, Pagès JM (2012). Broad-specificity efflux pumps and their role in multidrug resistance of Gram-negative bacteria. FEMS Microbiology Reviews.

[b91] Nilsson AI, Zorzet A, Kanth A, Dahlström S, Berg OG, Andersson DI (2006). Reducing the fitness cost of antibiotic resistance by amplification of initiator tRNA genes. Proceedings of the National Academy of Sciences of the United States of America.

[b92] Pakula AA, Sauer RT (1989). Genetic analysis of protein stability and function. Annual Review of Genetics.

[b93] Palmer AC, Kishony R (2013). Understanding, predicting and manipulating the genotypic evolution of antibiotic resistance. Nature Reviews Genetics.

[b94] Paulander W, Maisnier-Patin S, Andersson DI (2009). The fitness cost of streptomycin resistance depends on *rpsL* mutation, carbon source and *rpoS* (σ(s)). Genetics.

[b95] Perkins AE, Nicholson WL (2008). Uncovering new metabolic capabilities of *Bacillus subtilis* using phenotype profiling of rifampin-resistant *rpoB* mutants. Journal of Bacteriology.

[b96] Perron GG, Hall AR, Buckling A (2010). Hypermutability and compensatory adaptation in antibiotic-resistant bacteria. American Naturalist.

[b97] Petersen A, Aarestrup FM, Olsen JE (2009). The *in vitro* fitness cost of antimicrobial resistance in *Escherichia coli* varies with the growth conditions. FEMS Microbiology Letters.

[b98] Pfeiffer T, Schuster S, Bonhoeffer S (2001). Cooperation and competition in the evolution of ATP-producing pathways. Science.

[b99] Piddock LJ (2006). Multidrug-resistance efflux pumps – not just for resistance. Nature Reviews Microbiology.

[b100] Proshkin S, Rahmouni AR, Mironov A, Nudler E (2010). Cooperation between translating ribosomes and RNA polymerase in transcription elongation. Science.

[b101] Reynolds MG (2000). Compensatory evolution in rifampin-resistant *Escherichia coli*. Genetics.

[b102] Rodríguez-Verdugo A, Gaut BS, Tenaillon O (2013). Evolution of *Escherichia coli* rifampicin resistance in an antibiotic-free environment during thermal stress. BMC Evolutionary Biology.

[b103] Rozen DE, McGee L, Levin BR, Klugman KP (2007). Fitness costs of fluoroquinolone resistance in *Streptococcus pneumoniae*. Antimicrobial Agents and Chemotherapy.

[b104] Ruusala T, Andersson D, Ehrenberg M, Kurland CG (1984). Hyper-accurate ribosomes inhibit growth. The EMBO Journal.

[b105] Salverda ML, Dellus E, Gorter FA, Debets AJ, van der Oost J, Hoekstra RF, Tawfik DS (2011). Initial mutations direct alternative pathways of protein evolution. PLoS Genetics.

[b106] San Millan A, Heilbron K, MacLean RC (2014). Positive epistasis between co-infecting plasmids promotes plasmid survival in bacterial populations. The ISME Journal.

[b107] Schechner V, Temkin E, Harbarth S, Carmeli Y, Schwaber MJ (2013). Epidemiological interpretation of studies examining the effect of antibiotic usage on resistance. Clinical Microbiology Reviews.

[b108] Schenk MF, Szendro IG, Salverda ML, Krug J, de Visser JAGM (2014). Patterns of epistasis between beneficial mutations in an antibiotic resistance gene. Molecular Biology and Evolution.

[b109] Schrag SJ, Perrot V, Levin BR (1997). Adaptation to the fitness costs of antibiotic resistance in *Escherichia coli*. Proceedings of the Royal Society of London. Series B: Biological Sciences.

[b110] Seppälä H, Klaukka T, Vuopio-Varkila J, Muotiala A, Helenius H, Lager K, Huovinen P (1997). The effect of changes in the consumption of macrolide antibiotics on erythromycin resistance in group A streptococci in Finland. Finnish Study Group for Antimicrobial Resistance. New England Journal of Medicine.

[b111] Sezonov G, Joseleau-Petit D, D'Ari R (2007). *Escherichia coli* physiology in Luria-Bertani broth. Journal of Bacteriology.

[b112] Shcherbakov D, Akbergenov R, Matt T, Sander P, Andersson DI, Böttger EC (2010). Directed mutagenesis of *Mycobacterium smegmatis* 16S rRNA to reconstruct the *in vivo* evolution of aminoglycoside resistance in *Mycobacterium tuberculosis*. Molecular Microbiology.

[b113] Silva RF, Mendonça SC, Carvalho LM, Reis AM, Gordo I, Trindade S, Dionisio F (2011). Pervasive sign epistasis between conjugative plasmids and drug-resistance chromosomal mutations. PLoS Genetics.

[b114] Smith R, Coast J (2013). The true cost of antimicrobial resistance. British Medical Journal.

[b115] Snitkin ES, Zelazny AM, Thomas PJ, Stock F, Henderson DK, Palmore TN, NISC Comparative Sequencing Program (2012). Tracking a hospital outbreak of carbapenem-resistant *Klebsiella pneumoniae* with whole-genome sequencing. Science Translational Medicine.

[b116] Stoebel DM, Dean AM, Dykhuizen DE (2008). The cost of expression of *Escherichia coli* lac operon proteins is in the process, not in the products. Genetics.

[b117] Sundqvist M, Geli P, Andersson DI, Sjölund-Karlsson M, Runehagen A, Cars H, Abelson-Storby K (2010). Little evidence for reversibility of trimethoprim resistance after a drastic reduction in trimethoprim use. Journal of Antimicrobial Chemotherapy.

[b118] Tenaillon O, Rodríguez-Verdugo A, Gaut RL, McDonald P, Bennett AF, Long AD, Gaut BS (2012). The molecular diversity of adaptive convergence. Science.

[b119] Tokuriki N, Stricher F, Schymkowitz J, Serrano L, Tawfik DS (2007). The stability effects of protein mutations appear to be universally distributed. Journal of Molecular Biology.

[b120] Trindade S, Sousa A, Xavier KB, Dionisio F, Ferreira MG, Gordo I (2009). Positive epistasis drives the acquisition of multidrug resistance. PLoS Genetics.

[b121] Trindade S, Sousa A, Gordo I (2012). Antibiotic resistance and stress in the light of Fisher's model. Evolution.

[b122] Trinh V, Langelier MF, Archambault J, Coulombe B (2006). Structural perspective on mutations affecting the function of multisubunit RNA polymerases. Microbiology and Molecular Biology Reviews.

[b123] de Visser JA, Cooper TF, Elena SF (2011). The causes of epistasis. Proceedings of the Royal Society B: Biological Sciences.

[b125] de Vos M, Müller B, Borrell S, Black PA, van Helden PD, Warren RM, Gagneux S (2013). Putative compensatory mutations in the *rpoC* gene of rifampin-resistant *Mycobacterium tuberculosis* are associated with ongoing transmission. Antimicrobial Agents and Chemotherapy.

[b126] Walsh C (2000). Molecular mechanisms that confer antibacterial drug resistance. Nature.

[b127] Ward H, Perron GG, Maclean RC (2009). The cost of multiple drug resistance in *Pseudomonas aeruginosa*. Journal of Evolutionary Biology.

[b128] Weinreich DM, Delaney NF, DePristo MA, Hartl DL (2006). Darwinian evolution can follow only very few mutational paths to fitter proteins. Science.

[b129] Whiteley M, Lee KM, Greenberg EP (1999). Identification of genes controlled by quorum sensing in *Pseudomonas aeruginosa*. Proceedings of the National Academy of Sciences of the United States of America.

[b130] Wrande M, Roth JR, Hughes D (2008). Accumulation of mutants in “aging” bacterial colonies is due to growth under selection, not stress-induced mutagenesis. Proceedings of the National Academy of Sciences of the United States of America.

